# Degradation potentials of dissolved organic carbon (DOC) from thawed permafrost peat

**DOI:** 10.1038/srep45811

**Published:** 2017-04-05

**Authors:** Balathandayuthabani Panneer Selvam, Jean-François Lapierre, Francois Guillemette, Carolina Voigt, Richard E. Lamprecht, Christina Biasi, Torben R. Christensen, Pertti J. Martikainen, Martin Berggren

**Affiliations:** 1 Department of Physical Geography and Ecosystem Science, Lund University, Sweden; 2 Département de sciences biologiques, Université de Montréal, Canada; 3 Research Center for Watershed ― Aquatic Ecosystem Interactions (RIVE), Department of Environmental Sciences, Université du Québec à Trois-Rivières, Trois-Rivières, Québec, Canada; 4 Department of Environmental and Biological Sciences, University of Eastern Finland, Finland; 5 Arctic Research Centre, Aarhus University, Denmark

## Abstract

Global warming can substantially affect the export of dissolved organic carbon (DOC) from peat-permafrost to aquatic systems. The direct degradability of such peat-derived DOC, however, is poorly constrained because previous permafrost thaw studies have mainly addressed mineral soil catchments or DOC pools that have already been processed in surface waters. We incubated peat cores from a palsa mire to compare an active layer and an experimentally thawed permafrost layer with regard to DOC composition and degradation potentials of pore water DOC. Our results show that DOC from the thawed permafrost layer had high initial degradation potentials compared with DOC from the active layer. In fact, the DOC that showed the highest bio- and photo-degradability, respectively, originated in the thawed permafrost layer. Our study sheds new light on the DOC composition of peat-permafrost directly upon thaw and suggests that past estimates of carbon-dioxide emissions from thawed peat permafrost may be biased as they have overlooked the initial mineralization potential of the exported DOC.

Permafrost regions in northern latitudes store approximately 40% of the soil organic carbon globally^1^. These permanently frozen soils are vulnerable to increases in land surface temperature^2^ that cause deepening of the active layer (the upper soil layer which experiences seasonal thaw), thermokarst formation and a reduction in the geographical extent of permafrost^2^,^3^. Ecosystem and hydrology changes related to climate warming have been shown to increase the export of dissolved organic carbon (DOC) from permafrost catchments to recipient aquatic ecosystems in several regions^4^,^5^, although there are exceptions^6^,^7^. When this permafrost soil derived DOC reaches surface waters it becomes subject to different rates of processing by microbes and ultraviolet (UV) sunlight (bio- and photo-degradation, respectively), resulting in the production of greenhouse gases that ultimately escape to the atmosphere^8^. The return of even a small fraction of permafrost soil carbon to the atmosphere due to warming could thus have a significant impact on global carbon budgets.

High amounts of low molecular weight DOC with large potential for biological decay have been measured from >40,000 year old permafrost soil^9^. Furthermore, several studies have demonstrated, through measurements of different aspects of DOC processing, a high reactivity of DOC from thawed permafrost soils in aquatic environments. For example, a recent study demonstrated that ca 50% of >20,000 year old DOC from mineral permafrost soil was biologically degraded within a week of incubation at 20 °C^10^. Similarly, another study showed that a mean of 53% of >35,000 years old permafrost-derived DOC was biologically degraded within ca 8 days of incubation at 20 °C^11^. In Alaskan tundra catchments it was further established that a major fraction of the DOC that leaked into streams and lakes could be processed by UV light^12^. This processing either resulted in direct release of carbon dioxide (CO_2_) to the atmosphere or in the production of partially oxidized low molecular weight DOC compounds that, in turn, increased bacterial CO_2_ production rates^13^. There are thus several, direct and indirect pathways leading to an effective return of carbon to the atmosphere through exported DOC from permafrost soil upon thaw.

The above studies indicate that a substantial fraction of permafrost soil DOC may be either photo- or bio-degradable. However, most studies have assessed bio- and photo-reactivity separately or they have been conducted in mineral soil and not organic (peat) permafrost. Since Holocene northern peatlands are of special interest as they have acted as a long-term sink for atmospheric carbon^14^, containing presently ca 277 Pg of organic carbon in their deposits^15^, approximately equal to 33% of CO_2_-C in the atmosphere. As a result of global warming and changes in hydrology, peatlands have been found to experience a substantial loss of carbon due to increased CO_2_ release from decomposition, lower carbon accumulation rates^16^ and increased methane (CH_4_) emissions to the atmosphere^17^,^18^.

In the northern hemisphere, peatlands generally export larger amounts of DOC than forests^19^,^20^ and northern landscapes are rich in surface waters. Hence large amounts of DOC from wetland ecosystems could quickly reach aquatic ecosystems. Even though bio- and photo-degradation play a major role in DOC degradation in arctic aquatic ecosystems^10^,^12^, experimental evidence for the impact on these processes by permafrost peat thawing is still scarce. To our knowledge, no study has concurrently measured and compared the bio- and photo-degradability of peat-permafrost DOC with DOC from the active layer. Thus, we know only little on the coupling that exists between peat-permafrost and active layer DOC degradation potentials. Moreover, while previous studies have mainly focused on the CO_2_ production potentials from bacterial respiration (BR) based on permafrost thaw DOC^10^,^12^,^21^, the degree to which the permafrost soil DOC supports the production of new microbial biomass has been largely overlooked. There is thus a lack of integrated studies on the various facets of the initial reactivity of peat-permafrost DOC upon thaw that would allow understanding of the immediate fate of this DOC after it leaves the permafrost and before it reaches the aquatic environment.

Hence, the aim of this study was to simultaneously compare the bio- and photo-reactivity of the DOC extracted directly from experimentally thawed peat-permafrost with DOC derived from the overlaying active layer. We collected peat cores from a palsa mire in northern Finland and extracted soil pore water from five depths along the cores. To determine DOC composition, the soil solution extracted from the cores was analyzed fluorometrically and modeled using parallel factorial analysis (PARAFAC). We further incubated the soil pore water extracts to assess the bio- and photo-degradation of DOC, and the metabolic response of the natural bacterial assemblage.

## Results

### Optical properties of DOC in thawed permafrost layer and active layer

**Figure 1:**
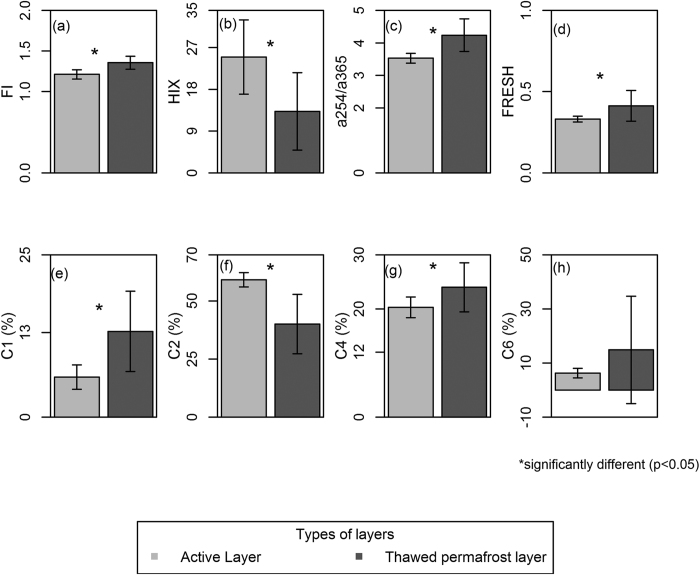
Dissolved organic carbon (DOC) composition in the active layer (n = 8) and the thawed permafrost layer (n = 8). The plots show (**a**) Fluorescece index (FI), (**b**) Humification index (HIX), (**c**) absorbtion ratio (a254/a365), (**d**) Freshness index (FRESH), (**e**) Humic-like fluorescence component (C1), (**f**) Humic-like fluorescence component (C2), (**g**) Humic-like fluorescence component (C4), and (**h**) protein-like fluorescent component (C6) are the DOC composition. Error bars indicate standard deviation of the mean. See section 2.1 for details of core collection and incubation.

We compared the optical characteristics of DOC originating from the active layer and the thawed permafrost layer to evaluate the potential effect of permafrost thaw on the DOC properties in receiving ecosystems. Overall, DOC originating from thawed permafrost exhibited different optical properties than that of the active layer, but the presence or absence of plant material had little effect (Supplementary Tables S1 and S2). For instance, the absorption ratio (a254/a365) was 18% higher in the thawed permafrost layer than the active layer (p = 0.001) while the humification index (HIX) was 61% higher in the active layer than in the thawed permafrost layer (p = 0.017, Fig. 1 and Supplementary Table S1). These patterns indicate that the DOC from the thawed permafrost layer has a higher proportion of low molecular weight DOC than the active layer. Coherent with the above metrics, fluorescence component C6, typically attributed to bio-reactive material, was proportionally higher in the thawed permafrost layer than in the active layer (Supplementary Table S1).

The freshness index (FRESH, p = 0.038) and the fluorescence index (FI, p = 0.002) were significantly higher in the thawed permafrost layer (FRESH = 0.41, FI = 1.36) than the active layer (FRESH = 0.33, FI = 1.21) (Fig. 1 and Supplementary Table S1). The humic-like fluorescence components C1 (p = 0.004) and C4 (p = 0.020) were 72% and 16%, respectively, higher in the thawed permafrost layer than in the active layer (Fig. 1e,g and Supplementary Table S1). On the contrary, the fluorescence component C2, which is also a humic-like substance, was 38% higher in the active layer than in the thawed permafrost layer (p = 0.003, Fig. 1f and Supplementary Table S1). The range of the slope ratio (S_R_) (0.62–0.88) and FI (1.14–1.47) values indicated that the DOC from the thawed permafrost layer and the active layer was of similar composition as the terrestrial DOC identified in most freshwater environments, as would be expected. Thus, although the DOC pools from both layers were representative of terrestrial DOC, the optical properties of thawed permafrost DOC more closely resembled freshly produced and highly degradable DOC found in freshwater systems compared with DOC found in the active layer (see discussion).

### Bio- and photo-degradability variation in thawed permafrost layer and active layer

**Figure 2:**
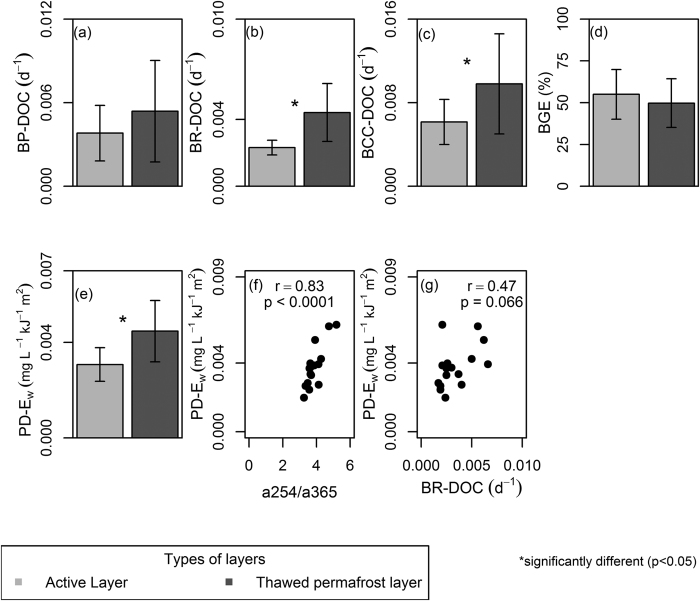
DOC degradation in the active layer (n = 8) and the thawed permafrost t layer (n = 8). For bio-degradation,the plots show (**a**) Bacterial production normalized to DOC (BP-DOC), (**b**) Bacterial respiration normalized to DOC (BR-DOC), (**c**) Bacterial carbon consumption normalized to DOC (BCC-DOC), and (**d**) Bacterial growth efficiency (BGE). In the plot (**e**) the DOC degradation rate per unit irradiation absorbed (PD-E_w_) is shown. Error bars indicate standard deviation of the mean. The plot (**f**) absorbtion ratio (a254/a365) vs PD-E_w_ (n = 16) and (**g**) BR-DOC vs PD-E_w_ (n = 16) are pearson correlation. See sections 2.3 and 2.4 for details of bio- and photo-degradation experiments, respectively.

The bacterial metabolic measurements agreed with the results on DOC optical properties and demonstrated a higher bio-degradability of the DOC extracted from the thawed permafrost layer than the active layer. Bacterial respiration (p = 0.005) and bacterial carbon consumption (p = 0.014) (normalized to DOC; BR-DOC and BCC-DOC) were 62% and 50% higher, respectively, in the thawed permafrost layer than in the active layer (Fig. 2 and Supplementary Table S1). However, bacterial production (normalized to DOC; BP-DOC) and bacterial growth efficiency (BGE) showed no significant differences between the thawed permafrost layer and active layer (Fig. 2 and Supplementary Table S1).

The photo-degradability of DOC (DOC degradation rate per unit irradiation absorbed [PD-E_w_]) was also 37% higher in the thawed permafrost layer than the active layer (p = 0.015, Fig. 2e and Supplementary Table S1). These results indicate that permafrost thaw may release a DOC pool that is both highly bio- and photo-degradable, exceeding the corresponding degradation potentials of DOC from the active layer.

## Discussion

This study provides an integrated representation of composition and degradation potentials of the DOC derived from thawed permafrost peat. We found that the initial degradability of the DOC from thawed permafrost layer is high compared to the DOC derived from active layer, suggesting that a substantial part of that DOC is sensitive to degradation before reaching surface waters. Furthermore, concurrent analysis of the bio-and photo-degradation of DOC derived from the thawed permafrost layer, provides insight into the DOC degradation potential at the interface where DOC is mobilized upon permafrost thawing.

### Photo-degradation of DOC in peat permafrost layer and active layer

Interestingly, the active layer contained higher shares of the humic-like fluorescence component C2 than the thawed permafrost layer (Fig. 1), a property that is typically associated with highly aromatic compounds^22^ and hence high photochemical reactivity^23^. The active layer DOC, however, had lower PD-E_w_ than the thawed permafrost layer. This is likely coupled to the presence of significantly higher humic-like fluorescence component C1 in the thawed permafrost layer than in the active layer. The component C1 has previously been found to be correlated with chemical compounds with higher aromaticity and higher molecular weight carbon than component C2^22^. Our results are important because the sunlight duration in the arctic and boreal zones is long during summer and the abundant thermokarst lakes are shallow^24^. Hence sunlight may penetrate the whole water column and degradation of this photo-reactive DOC could happen over most part of the day, once the DOC reaches the surface waters from thawing permafrost catchments. Thus, the high degradability measured in our study should translate into high processing rates in natural environments.

### Bio-degradation of DOC in peat permafrost layer and active layer

The DOC from thawed permafrost layer also showed higher biological degradability–both in terms of BR-DOC and BCC-DOC than that from the active layer, coherent with higher a254/a365 ratio and FRESH index. However, the contribution to bacterial growth (BP-DOC and BGE) was similar in the DOC measured for the different layers. This suggests that DOC released from the thawed permafrost peat would increase CO_2_ production and subsequent emissions, but it may not necessarily affect carbon, energy and nutrient transfer through bacterial biomass in aquatic food webs. This is in contrast to the pattern found in Imnavait Creek watershed on the North Slope of Alaska^25^, i.e. where BP-DOC and BGE were higher for DOM from the thawed mineral layer than for the active layer (organic soil) DOC. The Alaskan study, however, conducted the bacterial incubations without control on nutrients^25^. Nutrient limitation and temperature generally affect the bio-degradation of DOC^26^. Specifically, high concentrations of inorganic nutrients in combination with low incubation temperatures (e.g., as applied by ref. 25) result in high BGE values due to an interaction effect between temperature and nutrients^27^. The effect of nutrients alone, however, appears contentious as other studies have found no systematic effects of nutrient addition on DOC degradability in Arctic runoff^28^,^29^. Our incubations, which were conducted at a standard temperature and with control on nutrients to explore the inherent degradability of the DOC, revealed no significant difference in BP-DOC and BGE, respectively, between DOC from the thawed permafrost and the active layer.

### Effect of peat genesis and decomposition on DOC degradation potentials

Overall, the DOC from the thawed permafrost layer showed higher bio- and photo-degradability than the DOC derived from the active layer, which is likely due to differences in DOC composition, in turn related to permafrost history, vegetation and peat decomposition^30^. In palsa mires, as generally in peatlands, the origin of the peat differs at various depths due to moisture condition and plant communities changing with peatland development^31^,^32^. Rich wet fens occurred during the first permafrost aggradation (between 2000 and 3000 yr BP^33^) which are usually characterized by higher nutrient content and lower C/N ratios^34^. Within this wet ecosystem peat humification was likely inhibited, as contrasted to in the dry palsa ecosystems where peat in the active layer is highly humified shown by the significantly higher HIX in the active layer than in the permafrost. The differences in the peat genesis and decomposition, therefore, is of the possible reasons for the difference in the DOC composition and lability between the thawed permafrost layer and the active layer.

### Evidence of DOC bio-lability increase due to peat permafrost thawing

Studies have shown that the DOC from permafrost soil could be highly bio-labile when reaching nearby aquatic ecosystems^9^,^10^,^21^,^29^,^35^,^36^. Contrastingly, our study reports a DOC pool that is of relatively low bio-lability compared to reports from other studies. For example, our results show that ca 3% of the DOC was mineralized within a week of incubation which is similar to the findings in some permafrost and non-permafrost wetlands^37^,^38^, but much lower than what most others studies have reported in wetland catchments^9^,^10^,^21^,^29^,^35^,^36^. These differences in DOC biodegradability are likely explained by local differences in catchment characteristics^39^ and thus in DOC composition^30^,^39^. Regardless of regional differences in bulk DOC degradability, our results suggest that leaching of DOC from the thawed permafrost layer in northern Finland would increase the lability of the DOC pools in receiving aquatic ecosystems, as could be expected also in other regions with thawing peat permafrost^10^,^21^. Together with changes in biomass, wildfire, temperature and hydrology, the permafrost degradation could contribute to strongly altered export, composition and degradability of DOC in the coming decades^40^.

### Can protein like substance photo-degradable?

In boreal watersheds, a recent study found that bio- and photo-degradable DOC shared common origins but that bio- and photodegradable fractions were associated with different DOC pools across lakes, rivers and wetlands^45^. Our results suggest that thawed peat layer supplies a pool of DOC that is at the same time more bio- and photo-degradable, yet the composition is more akin to be described as bio- than photo-degradable material, i.e. protein-like and of low molecular weight. Does this indicate that a protein-like, low molecular weight DOC can also be highly photo-degradable if it has not yet been exposed to sunlight? The marginally significant positive correlation (p = 0.07) between the BR-DOC and PD-E_w_ might suggest that the pools of DOC that support bio- and photo-degradation could be the same (Fig. 2g), as further supported by the positive correlation between a254/a365 and PD-E_w_ (p < 0.0001) (Fig. 2f). We cannot rule out, however, that there are different pools of DOC originating from the thawed permafrost layer contributing to bio- and photo-degradation. The optical properties of the thawed permafrost layer DOC could be dominated by a protein-like signature while a smaller high molecular weight fraction may still be the main substrate for photochemical degradation. Further studies are needed to elucidate the mechanisms behind such patterns, nonetheless, our results emphasize an unexpected co-occurrence between DOC biological and photochemical degradability upon thawing of peat-permafrost.

### Conclusion and implications

The concurrent analysis of the various facets of DOC composition and degradability showed that the initial degradability of this DOC fraction was higher in the thawed permafrost layer than in the active layer, suggesting an efficient loss on its way to the receiving aquatic ecosystems. Therefore, the estimated lability (bio- and photo-degradation) of DOC from permafrost soil probably would have been higher in previous studies if they had included the initial degradability of DOC. Based on our results, a substantial labile fraction would be lost directly upon thaw, before or immediately entering the aquatic environment. While our study measured the potential for release, short-term labile DOC fractions such as organic acids are also known to be efficiently fermented into CH_4_ under anaerobic conditions that may occur in soils^41^.

As peatlands are important DOC sources to aquatic ecosystems^19^ and are known to be susceptible to changes in climate, hydrology and fire regimes^4^,^42^, our results suggest a potentially changing delivery of highly degradable DOC to surface waters in the face of future environmental changes. Our palsa study site which is located in the discontinuous permafrost zone^32^, will most likely be affected by global warming due to the weakening of thermal boundaries^43^ and result in extensive thawing and palsa collapse as reported in Northern Scandinavia^44^. Permafrost zones in northern latitudes are rich in surface waters^45^,^46^,^47^ and in the discontinuous permafrost zone the areal extent of surface waters is larger than either in the continuous or isolated permafrost zones^24^. Hence, this study suggests that the reactive DOC that is released from discontinuous peat-permafrost catchments will get degraded and released to the atmosphere as CO_2_ even more rapidly than the DOC exported from peatlands in the continuous and isolated permafrost zones once it reaches surface waters.

## Methods

### Study Site

Eight permafrost peat cores were collected from Peera Palsa (68°89′N, 21°05′E), which is a palsa mire near Kilpisjärvi. Intact peat profiles including living plants were collected at the end of September 2012; four cores from dry and four from natural bare peat surfaces. Coring was performed using a 80 cm long steel corer with exchangeable inner plastic tubes (diameter of 10 cm), which was hammered into the soil with a mechanical drill down to a depth of 80 cm. Immediately after sampling, peat cores were transported in mild freezing temperatures (−4 ± 2 °C) and subsequently stored at the same temperature from October 2012 to the end of March 2013. From the beginning of March 2013, the impermeable sealed peat cores were submerged into a ~−4 °C cold salt water bath to keep the peat cores under frozen conditions without any physical contact between the cores and the surrounding saltwater. The saltwater bath was located in a climate controlled chamber which had an air temperature of 10 °C. This study was part and made use of the set-up of a larger study that investigated the effect of sequential thawing on carbon and nitrogen cycling from subarctic peatlands. Hence, for a total time of 7 months the saltwater level was periodically lowered to increase the thawed (unfrozen) layer of the soil cores and at the end of 7 months, 20–40 ml of water was extracted from five depths along each core. Overall, the active layer in our experiment was unfrozen during a period of approximately twice the length compared to ambient conditions in northern Finland. However, assuming steady state conditions^48^ the extended length of the simulated unfrozen season should not affect DOC quantity and composition.

The depths we used to extract water were 5 cm, 20 cm, 40 cm, ‘AL-10cm’ (10 cm above the active layer depth) and ‘AL + 5 cm’ (5 cm below the active layer depth, i.e. permafrost) in accordance with the thawing steps used (see Supplementary Fig. S1). However, samples were finally pooled into two main sample groups, “active layer” (including all four active layer samples) and “thawed permafrost layer” (AL + 5 cm). (For more details on the study site, see Supplementary Methods). DOC concentrations of the water extracts were measured using a Shimadzu TOC analyzer 5000 (Kyoto, Japan). We further measured light absorption by the DOC from 200 nm to 800 nm using a Shimadzu UV-2600, UV-VIS spectrophotometer. Absorbance was converted to decadic absorption coefficient (α) by dividing the absorbance with the path length of the cuvette^49^. The α was in the range of 1.2 to 8.7 cm^−1^ at 254 nm, indicating highly colored samples. Hence all the samples were diluted to α of 0.5 cm^−1^ using deionized water. These diluted samples were used for conducting optical measurements, photo- and bio-degradation experiments.

### Optical DOC characterization

Excitation emission matrices (EEMs) were generated using a Cary Eclipse Fluorescence Spectrophotometer with excitation measured from 230 nm to 450 nm by 5 nm increments and emission from 260 nm to 600 nm by 2 nm increments. These EEMs were corrected for inner filter effect, instrument specific biases and normalized to the Raman water peak using the FDOM correct toolbox for MATLAB^50^. From the corrected dataset, the humification index (HIX)^51^, fluorescence index (FI)^52^ and freshness index (FRESH)^53^ were calculated. Further, we calculated absorption ratio at 254 nm and 365 nm (a254/a365)^54^, slope ratio (S_R_)^55^ and the DOC specific ultraviolet absorption at 254 nm (SUVA_254_)^56^, expressed as L mg C^−1^ m^−1^. One of the permafrost samples showed an extreme SUVA_254_ value of 8.90 L mg C^−1^ m^−1^ which is likely due to iron interferences^57^. We conducted a general linear model (GLM) test to assess the influence of this extreme value on the pattern between active layer and thawed permafrost layer and the statistical test showed that this extreme value affects the pattern substantially (Supplementary Table S2). Since we did not measure iron concentrations, we were not able to correct for this problem, hence we removed SUVA_254_ from the analysis.

Given the small number of samples, we quantified the fluorophores using a PARAFAC model that was developed for over 1300 boreal freshwater samples originating from lakes, rivers and wetlands with high terrestrial influence^58^. This model identified 6 fluorescence components, where components C1 to C5 were associated to humic-like substances and the component C6 was representative of freshly produced protein like substances^58^. (For more details of the modeling, see Supplementary Methods). Fluorescence spectroscopy only characterizes a fraction of the DOC sample, but nonetheless it captures broad families of molecules as revealed by comparisons with the output from other techniques such as FTIRC-MS^22^,^59^. Hence we used fluorescence as a proxy of DOC composition as done by others^22^,^58^,^60^,^61^,^62^.

### Photochemical Degradation

The photochemical degradation has been explained in detail elsewhere^63^. Briefly, the water samples were filtered using 0.45-μm filter (30 mm PES filters, Thermo scientific, MA, USA); 10 ml of the sample was filled in a 20 ml quartz vial and the remaining 10 ml of the headspace was flushed with standard synthetic air mixture and sealed with butyl rubber septa. For each sample, four vials were filled with filtered water, and two vials were kept as control in which 0.1 ml of concentrated H_3_PO_4_ was added immediately. The other two vials were kept horizontally on a spinning disc (0.67 rpm) to ensure a uniform light dose. The vials were placed 40 cm below two Sodium-Xenon lamps (SON-T AGRO 400, Idman Philips Lighting, NL) at 20 ± 1 °C for two days in a climate chamber (Model:BDR 16, Conviron, Winnipeg, MB, Canada). The UV irradiation at different parts of the disc ranged from 3.64–6.89 Wm^−2^ for UV-A and 0.06–0.1 Wm^−2^ for UV-B (Spectroradiometry, International Light Inc), which is roughly equal to the mean day-time radiation level during summer in Southern Sweden.

After irradiation, 0.1 ml of concentrated H_3_PO_4_ was added to convert DIC to CO_2_, shaken for one minute, and then left 4–5 hours to attain equilibrium. After equilibrium, CO_2_ in the headspace was measured using an EGM-4 infrared gas analyzer (PP Systems, Amesbury, MA, USA). We assumed that if bacteria survived the UV dose, which is unlikely, the DIC produced by BR could not significantly influence photo-degradation rates since these rates measured over 2 days were at least 17 times greater than BR.

The DOC degradation rate per unit irradiation absorbed (PD-E_w_) was calculated to know the quantity of DOC degraded per unit of irradiation energy absorbed. The total absorbed energy (E_w_) calculation has been explained in detail elsewhere^64^. Briefly, the Ew was calculated based on lamp energy, incubation time and the amount of energy absorbed by each samples at each wavelength from 300 nm to 550 nm.


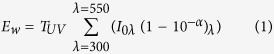



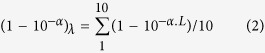


T_UV_ is the incubation time in seconds, I_0λ_ is the lamp energy in Wm^−2^, (1 − 10^−α^)_λ_ is the mean irradiation absorbed by the samples at a corresponding wavelength (λ). In order to calculate 1 − 10^−α^, the incubated vial was split horizontally into ten different sections to account for the average irradiation path length of different sections of the vial at each wavelength (Eq. 2). The L is the mean irradiation path length in each cross section of the vial (cm).

### Bacterial Metabolism

Water samples for potential bacterial metabolism were incubated with native bacterial communities in 25 ml glass vials under dark conditions at 20 °C for seven days. Inorganic nutrient deficiencies could affect the bio-degradation of DOC, hence 50 μg L^−1^ of P and 500 μg L^−1^ of N were added at the beginning of the incubation. Bacterial production (BP) was measured at different time intervals at day 1, 3 and 7 using the ^3^H-leucine incorporation method^65^. Bacterial respiration (BR) was assessed by measuring oxygen consumption over time using non-invasive optical oxygen sensors (Presens GmbH, Regensburg, Germany). The slope of linear regression line for oxygen *vs* time was used to calculate the DOC consumption, assuming a respiratory quotient (RQ) of 1 which is a standard assumption and well constrained within the range of RQ in soils^66^. The BP and BR were used to calculate the bacterial carbon consumption (BCC = BP + BR) and the bacterial growth efficiency (BGE = BP/[BP + BR]). In order to emphasize the qualitative nature of DOC bio-degradability, here we normalized BP, BR and BCC to DOC concentrations and hereby referred to as BP-DOC, BR-DOC and BCC-DOC, respectively.

### Statistical Analyses

The data collected from the peat cores represented two different factors (active layer or thawed peat layer; with or without vegetation) and several dependent variables (BP-DOC, BR-DOC, BCC-DOC, BGE, PD-E_w_, FI, HIX, FRESH, a254/a365, C1, C2, C4, C5, C6 and S_R_). We used a general linear model (GLM) to test the significance of the different treatments. Data with skewed distribution (skew >2) were log transformed before GLM analysis. GLM test was conducted in IBM SPSS 22 (IBM Corp., Armonk, NY, USA).

## Additional Information

**How to cite this article:** Panneer Selvam, B. *et al*. Degradation potentials of dissolved organic carbon (DOC) from thawed permafrost peat. *Sci. Rep.* **7**, 45811; doi: 10.1038/srep45811 (2017).

**Publisher's note:** Springer Nature remains neutral with regard to jurisdictional claims in published maps and institutional affiliations.

## Supplementary Material

Supplementary Information
