# Supplementary material for: Degradation potentials of dissolved organic carbon (DOC) from thawed permafrost peat
Source: Sci Rep. 2017 Apr 5;7:45811. doi: 10.1038/srep45811 (PMC5395014; doi:10.1038/srep45811)
Supplement: Supplementary Information [file srep45811-s1.pdf]

Supplementary information for "Degradation potentials of dissolved organic carbon (DOC) from thawed permafrost peat"

Balathandayuthabani Panneer Selvam[1*], Jean-François Lapierre[2], Francois Guillemette[3], Carolina Voigt[4], Richard E. Lamprecht[4], Christina Biasi[4], Torben R. Christensen[1, 5], Pertti J. Martikainen[4], and Martin Berggren[1]

[1]*Department of Physical Geography and Ecosystem Science, Lund University, Sweden.*

[2]*Département de sciences biologiques, Université de Montréal, Canada.*

[3]*Research Center for Watershed–Aquatic Ecosystem Interactions (RIVE), Department of Environmental Sciences, Université du Québec à Trois-Rivières, Trois-Rivières, Québec, Canada.*

[4]*Department of Environmental and Biological Sciences, University of Eastern Finland, Finland.*

[5]*Arctic Research Centre, Aarhus University, Denmark.*

Supplementary Methods

Study site

A palsa is defined as peat which is lifted above the surrounding mire by permafrost[1]. The palsa in our study site rises ca. 3 m above the surrounding peat and it is at the starting stage of collapse and classified as a dome shaped and peat-cored palsa[2-4]. The ice content in the permafrost is likely high as peat cored palsas in northern Finland usually consist of peat that is perenially frozen and includes ice crystals in the peat pores and with segregated ice formation[5]. Palsas are a characteristic of the discontinuous permafrost zone[6] and most of the palsa mires in northern Finland are <1000 years old[7] or at most 2000-3000 years old in northernmost Finland[8]. The original peatland developed far earlier, ca. 8000-9000 years BP[8]. The peat on the surface is originating from Bryales mosses, lichens and Ericales shrubs with different origin at depth, e.g. consisting of Sphagnum, Carex and Eriophorum[2].

Intact peat profiles including living plants were collected at the end of September 2012 when annual thaw depth was at its maximum and the average active layer (AL) was 65 cm. Four cores from dry parts of the palsa mire were sampled, which are sparsely vegetated with dwarf shrubs such as *Empetrum hermaphroditum* and *Vaccinium vitis-idaea*, covered by brown mosses as well as lichen species commonly found on palsa surfaces[5,8-10]. Additionally, four cores were collected from natural bare peat surfaces (Figure S1). Batches of bare peat surfaces occur among the vegetated ones, mainly due to wind abrasion[11]. Coring was performed using a 80 cm long steel corer with exchangeable inner plastic tubes (diameter of 10 cm), which was hammered into the soil with a mechanical drill down to a depth of about 80 cm. Immediately after sampling, peat cores (containing about 65 cm of active layer and 15 cm of permafrost) were transported in mild freezing temperatures (-4°C ± 2) and subsequently stored at the same temperature from October 2012 to the end of March 2013.

In the beginning of March 2013, the impermeable sealed peat cores were incubated by setting them in an upright position in a water bath. The water bath was filled with salt water to keep the peat cores under frozen conditions, as the saltwater had a temperature of around -3/-4°C. There peat cores were not in physical contact with the salt water. This set-up was arranged in a climate chamber with an adjusted air temperature of 10°C. This study was part and made use of the set-up of a larger study that investigated the effect of sequential thawing on carbon and nitrogen cycling from subarctic peatlands. From an initially frozen state (-4°C), the cores were thawed in four-week steps, by lowering the salt-water level and thus increasingly exposing them to a constant air temperature of 10 °C. In the last experimental phase after 7 months the full core profile, including the permafrost part, were unfrozen (Figure S2). At that stage 20-40 ml of water were extracted via sampling outlets using a syringe with a Luer Lock Tip (Terumo®) from five depths. This experimental set-up was intended to simulate palsa collapse and to mimic the effect of an unusual warm and wet summer on biogeochemical cycles. Hence the water table level inside the cores was artificially raised and kept constant at 5-10 cm below the surface by adding milli-Q water.

In order to simulate the natural state and to make our study comparable to field conditions, the peat cores were kept under as close to natural conditions as possible during the treatment and transport and storage period.

Optical DOC characterization

Given the small number of samples, we quantified the fluorophores using a PARAFAC model that was developed for over 1300 boreal freshwater samples originating from lakes, rivers and wetlands with high terrestrial influence[12]. This model has been used to study the patterns in bio- and photo-degradation of DOC in a wide number of systems. Further, this model identified 6 fluorescence components that have been associated to detailed chemical characterization in a subset of boreal rivers using high resolution mass spectrometry[13]. In particular, components C1 to C5 were associated to a diverse set of humic-like substances and the component C6 was representative of freshly produced protein like substances[12]. The component C3 has been associated to high photochemical reactivity[12] but it was absent in most of our samples.

References

1 Seppälä, M. The term ‘palsa’. *Z. Geomorphol.* **16**, 463 (1972).
2 Seppälä, M. in *Advances in periglacial geomorphology* (ed M.J. Clark) 247-278 (John Wiley, 1988).
3 Gurney, S. D. Aspects of the genesis, geomorphology and terminology of palsas: perennial cryogenic mounds. *Prog. Phys. Geogr.* **25**, 249-260, doi:10.1177/030913330102500205 (2001).
4 Hofgaard, A. Effects of climate change on the distribution and developments of palsa peatlands: background and suggestions for a national monitoring project. Report No. 952-11-2296-X, 33 (Norwegian Institute for Nature Research Project, Norway, 2003).
5 Seppälä, M. in *Finland - land of mires* (eds T Lindholm & R Heikkilä) 155-162 (Finnish Environment institute, 2006).
6 Seppälä, M. Introduction to the periglacial environment in Finland. *Bulletin of the Geological Society of Finland* **69**, 73-86 (1997).
7 Seppälä, M. Dating of palsas. *Geological Survey of Finland, Special Paper* **40**, 79-84 (2005).
8 Oksanen, P. Holocene development of the Vaisjeäggi palsa mire, Finnish Lapland. *Boreas* **35**, 81-95, doi:10.1080/03009480500359103 (2006).
9 Oksanen, P. O., Kuhry, P. & Alekseeva, R. N. Holocene development of the Rogovaya River peat plateau, European Russian Arctic. *Holocene* **11**, 25-40, doi:10.1191/095968301675477157 (2001).
10 Oksanen, P. O., Kuhry, P. & Alekseeva, R. N. Holocene Development and Permafrost History of the Usinsk Mire, Northeast European Russia. *Geogr. Phys. Quatern.* **57**, 169, doi:10.7202/011312ar (2003).
11 Marushchak, M. E. *et al.* Hot spots for nitrous oxide emissions found in different types of permafrost peatlands. *Global Change Biol.* **17**, 2601-2614, doi:10.1111/j.1365-2486.2011.02442.x (2011).
12 Lapierre, J. F. & del Giorgio, P. A. Partial coupling and differential regulation of biologically and photochemically labile dissolved organic carbon across boreal aquatic networks. *Biogeosciences* **11**, 5969-5985, doi:10.5194/bg-11-5969-2014 (2014).

13 Stubbins, A. *et al.* What's in an EEM? Molecular signatures associated with dissolved organic fluorescence in boreal Canada. *Environ. Sci. Technol.* **48**, 10598–10606, doi:10.1021/es502086e (2014).

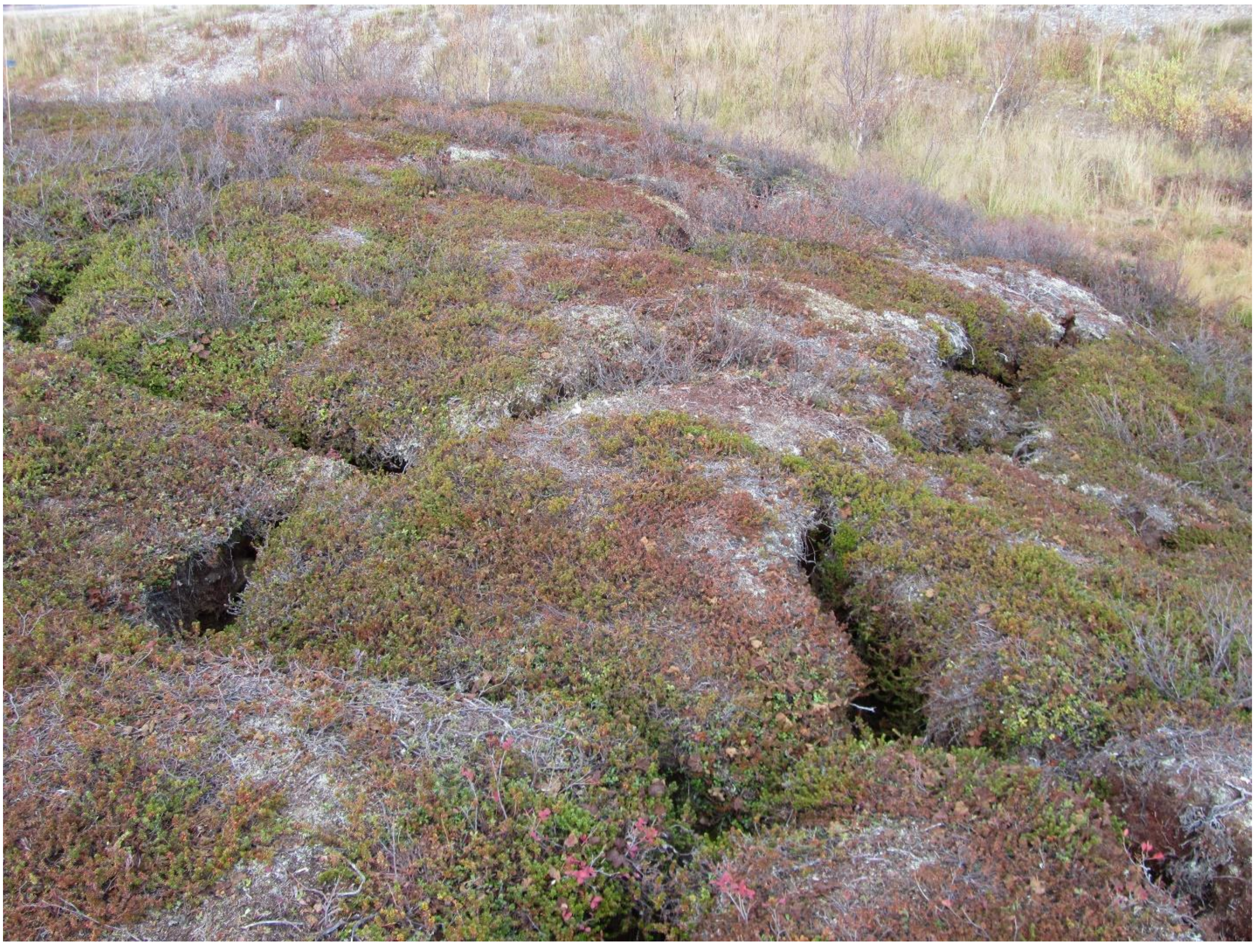

Figure S1. Permafrost peat site where we collected samples. Four cores from dry and four cores from natural bare peat surfaces were collected.

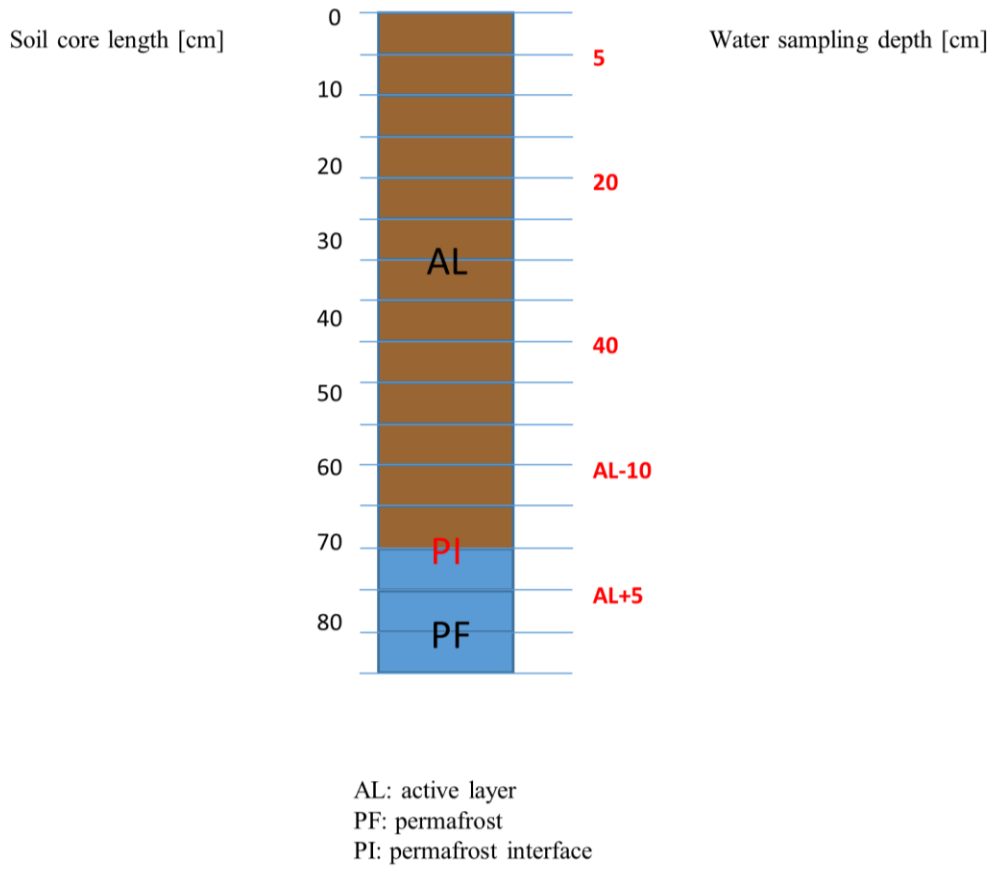

Figure S2. Soil core length and water sampling depth in the soil cores. The depths we used were 5cm, 20cm, 40cm, AL-10cm (active layer) and AL+5cm (permafrost) in accordance with the thawing steps used.

Table S1. General linear model was performed to test the influence of soil layers (active layer and permafrost) and vegetation (presence and absence) on DOC composition and degradation.

	Types of layers				Presence and absence of vegetation			
Variables	d.f.	MS	F	p	d.f.	MS	F	p
FI	1	0.08	14.54	.002*	1	0.00	0.03	.871
HIX	1	550.82	7.58	.017*	1	61.30	0.84	.376
FRESH	1	0.03	5.46	.038*	1	0.00	0.36	.561
BIX	1	0.02	4.75	.050	1	0.00	0.41	.535
a254/a365	1	2.00	18.54	.001*	1	0.35	3.28	.095
C1	1	198.53	12.67	.004*	1	59.75	3.81	.075
C2	1	1455.61	14.47	.003*	1	0.42	0.00	.950
C4	1	55.61	7.22	.020*	1	43.26	5.61	.035*
C5	1	0.20	0.02	.885	1	8.60	0.97	.345
C6	1	0.117	1.463	.25	1	0.15	1.87	.20
Sr	1	0.05	1.62	.228	1	0.03	0.90	.361
BP/DOC	1	0.00	2.55	.137	1	0.00	2.31	.155
BR/DOC	1	0.00	11.94	.005*	1	0.00	0.49	.498
BCC/DOC	1	0.00	8.22	.014*	1	0.00	2.27	.158
BGE	1	110.30	0.69	.421	1	162.50	1.02	.332
PD-E_w	1	0.00	8.02	.015*	1	0.00	2.27	.158

*significantly different ($p<0.05$)

Table S2. General linear model performed to test the influence of soil depth (active layer and permafrost) and vegetation (presence and absence) on $SUVA_{254}$. Results are shown for the complete data set and for a modified data set excluding an extreme $SUVA_{254}$ value of 8.90 L mg C^{-1} m^{-1}.

	Types of layers				Presence and absence of vegetation			
Variables	d.f.	MS	F	p	d.f.	MS	F	p
By including the extreme $SUVA_{254}$								
$SUVA_{254}$	1	0.24	0.18	0.68	1	7.63	5.74	0.03
By excluding the extreme $SUVA_{254}$								
$SUVA_{254}$	1	1.98	4.81	0.05	1	2.95	7.16	0.02
